# Improving access to free school meals: Evaluating the implementation of free school meal auto-enrolment processes

**DOI:** 10.1371/journal.pone.0339477

**Published:** 2026-02-17

**Authors:** Rob Oxley, Sundus Mahdi, Louise Padgett, Patience Gansallo, Myles Bremner, Cressida Pidgeon, Annie Connolly, Kate E. Pickett, Bob Doherty, Maria Bryant

**Affiliations:** 1 Department of Health Sciences, University of York, York, United Kingdom; 2 Bremner & Co, Kineton, Warks, United Kingdom; 3 The Trussell Trust, Salisbury, Wiltshire, United Kingdom; 4 School for Business and Society, University of York, York, United Kingdom; 5 Hull York Medical School and the Department of Health Sciences, University of York, York, United Kingdom; University College London, UNITED KINGDOM OF GREAT BRITAIN AND NORTHERN IRELAND

## Abstract

To receive benefits-related free school meals in England, households must currently have an annual household income of less than £7,400 (before any benefits-related income), and parents must submit an application. However, data estimates that ~11% do not apply. This equates to about 215,000 children who could, but do not, receive meals they are entitled to. As schools receive pupil premium funding based on these free school meal allocations, under registrations can impact children, families and the support that schools can offer. Free school meal auto-enrolment is a term to describe the processes by which local governments use welfare datasets to identify and register entitled children, allowing parents an opportunity to opt out should they wish. This process can increase registration uptake, though the current evidence regarding free school meal auto-enrolment is limited. This study aimed to explore factors that influence the successful implementation of free school meal auto-enrolment from a local government perspective. Qualitative data were collected through semi-structured interviews with local government authority representatives and national stakeholders (n = 20) across England, supported by documentary analysis (n = 142 relevant documents). Data were analysed deductively according to the Context and Implementation of Complex Interventions framework. Results revealed there was variation in how local governments undertook and experienced auto-enrolment processes, based on the degree to which it was prioritised, available resources and encountered barriers. Multiple barriers to implementation were described, including capacity, data access and resistance regarding data sharing legalities from information governance colleagues. While barriers could be overcome with adequate leadership support, funding and capacity, the reliance on these conditions may lead to inconsistent auto-enrolment delivery and a lottery in free school meal access across the country.

## Introduction

For eligible pupils, receiving free school meals (FSM) can reduce the financial burden on families, provide access to nutritious food and improve educational attainment [1,2]. Moreover, FSM uptake can aid schools in obtaining funding to support disadvantaged children, while increasing the need for school catering. FSM can consequently, lead to additional employment opportunities for school and supply chain staff, strengthening the local and wider community and driving cost savings through economies of scale [3].

According to government data, approximately 2.2 million pupils (25.7%) are eligible for FSM, a figure that has consistently increased since 2017 [4], and may be attributed to rising poverty, related to socioeconomic instability or transitional protection for FSM [5,6]. In the context of FSM, transitional protection ensures that any child that has become eligible for FSM from April 2018 will continue to be eligible until the end of the 2025/26 academic year, regardless of household income, and may consequently increase FSM eligibility. In addition, with the recent expansion of FSM eligibility to children in households receiving universal credit [7], eligibility may increase further in 2026. For the purposes of this paper, the term ‘entitled’ refers to children who reach the FSM eligibility threshold (and may or may not receive FSM), as distinct from ‘registered’, which refers to entitled children who currently receive FSM (also termed ‘eligible’ by the DfE).

At the time of writing, in the current system in England, FSM are means-tested and require a household income of <£7,400 to be entitled (before benefits-related income). If entitled, families are required to apply, in the majority of cases, to their local government (referred to as a local authority or ‘council’ in England); however, data cited by the central DfE state that approximately 161,400 (~11%) of those entitled do not submit applications [8]. More recent estimates suggest that this number is closer to 220,000 [9].

There are several reasons why families may not apply, including stigma, shame and administrative and technological burden (influenced by language, literacy and access barriers) [10,11]. This means families, who could save on school meal costs, are not doing so, and schools miss out on pupil premium funding; worth £1,515 per registered primary-aged child and £1,075 per registered secondary pupil [12]. For context, the Pupil Premium grant is separate from the core funding schools receive (calculated through the national funding formula), which is currently a minimum of £4,955 per primary pupil and £6,465 per secondary pupil [13]. Instead pupil premium is primarily allocated based on the number of pupils who are or have been (in the past 6 years) registered for free school meals. Notably, pupil premium funding is not exclusive to FSM pupils and is separate from school meal funding. Therefore, any pupil premium the school receives can be used to improve learning, enhance academic support and provide extracurricular activities. However, it is important to note that the purpose of pupil premium funding is to narrow the attainment gap between the most and least disadvantaged pupils, and how schools spend pupil premium is monitored by the government department, Ofsted [12].

FSM auto-enrolment is a term that describes the processes that ensure as many families as possible who are entitled to FSM receive them without having to directly apply to schools or local authorities. Instead, families who claim housing benefits or council tax support, but who have not applied for means-tested FSM, are identified by their local authority through searching relevant welfare databases. Identified, entitled families are sent letters to let them know that the council will apply on their behalf, but that they can withdraw if they do not wish this to happen. Those not opting out are then automatically registered to receive FSM and the school is provided with the additional associated pupil premium funding. This auto-enrolment differs from (and should be complementary with) policies that offer universal FSM, in which application processes for means-tested FSM remain an essential way to ensure that schools receive the associated pupil premium funding.

In 2021, the UK National Food Strategy recommended that central government ‘enrol eligible children for free school meals automatically’ [13–16]. Before this, a bill was proposed in 2015, with support from Members of Parliament (MPs) from all parties, though this was ultimately unsuccessful [17]. Despite these recommendations and previous discussions in parliament, the responsibility for implementing auto-enrolment remains with local authorities. However, there is currently limited evidence on implementing these processes from a local authority perspective.

Previous research has highlighted barriers to implementing meal-related processes and policies within school settings, such as increased staff workload and greater administrative burden to correspond with parents [18–20]. From a local authority perspective, similar research has also highlighted implementation facilitators to support the delivery of new school food policies or interventions, such as effective communication and ensuring sufficient staff capacity to do so [21–23]. Specifically, this research found that perceptions of relevant parties, including teachers, kitchen staff and local authority stakeholders, can highly influence the successful implementation of new policies. Learning from local authorities about FSM auto-enrolment processes can be indispensable to understanding the factors that facilitate, slow or block implementation, and may highlight implementation strategies.

Although there is limited academic literature on FSM auto-enrolment, some local authorities who have implemented the processes have shared information on the impact that it has had at a local level. For example, from 2016 when Sheffield City Council implemented FSM auto-enrolment (a process they termed ‘auto-award’) to 2024, the local authority identified an average of 700 additional pupils per year, equating to ~£500,000 in additional pupil premium funding provided to schools each year [24]. More recently, a report published by the Education Policy Institute highlighted similar impact, showing that two local authorities had identified an additional 600 and 275 children entitled to FSM respectively [25]. Despite this positive impact, the report crucially highlights challenges with setting up such a process, which vary between local authorities [25].

In 2023, our programme of research; FixOurFood in Schools’ worked in partnership with Sheffield City Council to develop and test resources to support other local governments to set up auto-enrolment processes. Given the potential impact on FSM registrations and funding, and the recognised barriers to the current self-registration method, there followed an increasing awareness and interest among local authorities and other organisations regarding the adoption of FSM auto-enrolment processes. One such organisation is the Local Government Association, who advocate for FSM auto-enrolment adoption [26]. Recently, FSM auto-enrolment was included in the House of Lords ‘Recipe for Health’ report [15] and was debated in the House of Commons [27].

As the policy environment continues to develop, with expansions to FSM entitlement and changes to transitional protection, numerous socioeconomic issues continue to impact the population. Over recent years, Brexit, COVID-19, a cost of living crisis and ongoing Russia-Ukraine conflict [6,28,29] have compounded to create a ‘polycrisis’, whereby simultaneous crises interact with one another, leading to more severe outcomes [30]. Combined, these conditions have led to job losses, reductions in household net income and increases in living costs [31,32]. Such issues disproportionately impact low-income households [29] and highlight the need for effective support. Moreover, the recent expansion of FSM entitlement further highlights the importance of such processes to ensure registration of all entitled pupils [7]. However, research exploring FSM registration processes, in different contexts, is currently lacking. Consequently, this evidence gap may lead to decisions that lack a robust evidence base.. As such, this research aimed to evaluate the implementation of FSM auto-enrolment from a local authority and national stakeholder perspective, including identifying the key processes, core elements and agents required to set up and deliver auto-enrolment within a local authority area and how these are influenced by the setting, context and implementation strategy. Whilst doing so, we also explored the potential barriers and facilitators to successful implementation.

## Methodology

### Study design

This action research was delivered within a larger programme of work called ‘FixOurFood’ [33], which aimed to transform the food system across Yorkshire, United Kingdom. Members of the FixOurFood team delivered webinars and workshops on FSM auto-enrolment to local authority and national stakeholders, aiming to co-design support for implementation.

Given the scale of FSM auto-enrolment implementation, areas outside of Yorkshire were included in this research. This research aimed to evaluate the implementation of FSM auto-enrolment processes through qualitative approaches, including semi-structured interviews with local authority representatives (*n* = 15) and national stakeholders (*n* = 5) across England, and a documentary analysis of relevant local government documents (*n* = 142). Such qualitative methods are appropriate when exploring implementation processes, barriers and facilitators, as they allow for in-depth investigation of how and why implementation may succeed or fail, from the perspective of those implementing the intervention and in the wider context in which the intervention is implemented [34].

This evaluation used interview and documentary analysis data to explore which (and how) aspects of context/setting interact with the implementation process, which (and how) local authorities employed different implementation strategies and which (and how) implementation agents are involved in implementation. Ethical approval for the study was obtained from the Department of Health Sciences Research Governance Committee at the University of York (Ref: HSRGC/2023/586/H).

### Setting, recruitment and sample

Recruitment began on the 29^th^ of September 2023 and ended on the 6^th^ of April 2025. As this project was embedded within the wider FixOurFood programme that focused on Yorkshire food systems, recruitment began in Yorkshire, England. Recruitment was not restricted to any region, however, due to the increasing awareness and interest in auto-enrolment, this project was expanded more widely.

Considering the aim of the study, local authority representatives were identified through existing contacts and were recruited using purposive and snowball sampling, to ensure participation from local authorities at various stages of implementation. National stakeholders were identified as those holding senior positions within school food-related organisations and were recruited through purposive sampling to ensure that, in the context of FSM auto-enrolment, the most relevant stakeholders were recruited. As those responsible for auto-enrolment implementation, local authority representatives could provide a first-hand account of the implementation processes, barriers and facilitators in their area. Given their involvement in school food more widely, and having worked with several local authorities, national stakeholders were able to discuss common processes, barriers and facilitators to implementation across the country. Those who expressed interest were sent a participant information sheet and interview invitation approximately one week before the interview via email. Informed consent was provided either prior to the interview (written) or verbal consent at the start of the interview, which was audio recorded.

A member of the research team screened local authority participants, who were eligible if they (i) expressed interest in participating in the research project and (ii) had set up, or were setting up FSM auto-enrolment processes in their area. National stakeholders were approached directly based on their involvement in FSM auto-enrolment.

### Data collection

#### Stakeholder interviews.

RO, SM and LP conducted interviews. Interviews were conducted online through Zoom [35] or Microsoft Teams [36]. If appropriate, interviews could have involved two stakeholders from the same local authority.

Interviews explored the context in which FSM auto-enrolment was implemented, alongside the factors that influenced implementation, implementation processes and barriers and facilitators. Interviews also focused on data protection, governance, workforce requirements and partnership with schools. Local authority topics varied slightly, based on the stage of auto-enrolment implementation (see S1 Appendix). Local authority and national stakeholder interview topics were broadly similar. However, the former focused primarily on local implementation, processes and barriers, while the latter included topics such as local vs national implementation and policy recommendations (see S1 and S2 Appendix). Demographic characteristics were also shared by participants during interviews to provide a contextual understanding of the stakeholders implementing auto-enrolment. In interviews with two participants, only the demographics of the primary participant are reported to maintain participant anonymity.

#### Document collection.

PG conducted the documentary analysis. Documentary analysis was used to explore factors that influence implementation in local authority areas [38,39]. Documents were gathered with permission from local authority stakeholders to support qualitative data analysis (including, but not exclusively those areas that took part in the interviews). For those that did not participate in interviews, information regarding the documentary analysis was shared in webinars and workshops, where local authority stakeholders were invited to share any relevant documents with the study team. Document sharing by interview participants was not compulsory. The type of documents included (but were not limited to) progress updates shared through emails (e.g., from directors and implementing stakeholders), workshop notes (e.g., from data support workshops), meeting minutes and other documents (e.g., data protection impact assessment [DPIA], briefs or business cases). Documents could be either printed or electronic and were collated over the 18-month study period (1st July 2023–31st December 2024). All documents were stored in a secured drive.

### Data analysis

**Fig 1:**
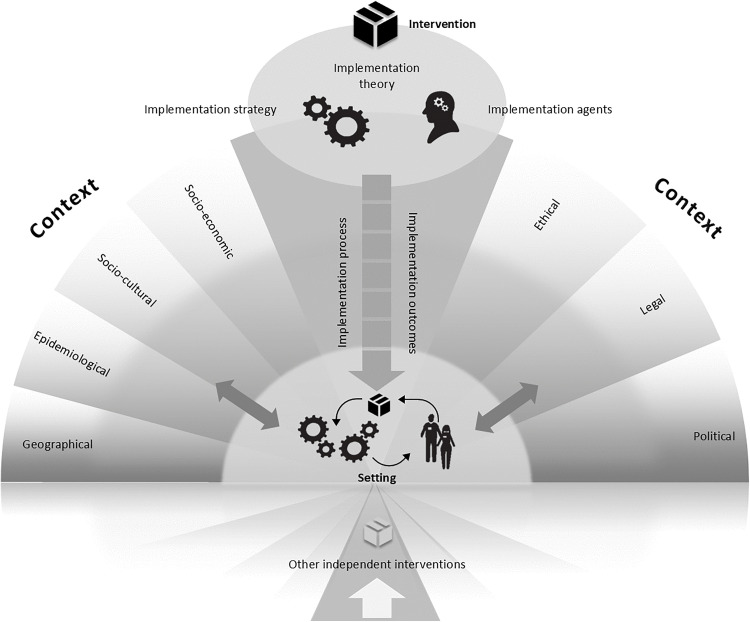
The context and implementation of complex interventions (CICI) framework comprising the three dimensions of context, implementation and setting [40]. Reprinted with permission.

Data were analysed according to the Context and Implementation of Complex Interventions (CICI) framework (Fig 1) [40], which provided a structure to analyse any data relating to the wider context in which FSM auto-enrolment was implemented. Specifically, this framework was used to understand the implementation of the auto-enrolment programme, within the wider system, which differs from other implementation-focused frameworks that do not consider these aspects The CICI framework also supported analysis of data regarding implementation processes, strategies and agents.

#### Interview data analysis.

Interview data were collected by members of the research team, then sent for transcription by a third party. Once returned, members of the research team proofread the transcripts to remove any identifiable information and to ensure that the transcripts provided an accurate account of the interview. Three transcripts were double coded by members of the research team, who then discussed and resolved discrepancies. Following this, a codebook based on framework constructs was developed, which was used for all future analyses.

Each transcript was coded deductively according to the CICI framework by one member of the research team. Where data did not fit within the framework, they were coded inductively and, if relevant, data were coded into multiple constructs. All data were coded using NVivo 14 software [37]. Following data coding, a framework matrix was developed using CICI constructs and sub-constructs, which was used to explore the data and develop themes. Findings are reported according to the overarching framework constructs and themes within these.

#### Documentary analysis.

An initial review was undertaken to understand the narrative of FSM auto-enrolment implementation, based exclusively on the documents gathered. Following this, documents were categorised based on the relevance of the document and the information provided. Data analysis was based on Braun and Clarke’s model of thematic analysis [41]. After a detailed review of the documents, deductive thematic analysis was undertaken according to the CICI framework, by one member of the research team. During coding, document quotes were logged chronologically to help observe changes over time. Each document was reviewed by researchers, with quotes, themes and interpretations jointly refined. A framework matrix was then developed, which categorised key findings into constructs and sub-constructs, based on the CICI framework.

## Results

### Stakeholder interviews

21 local authority representatives from 15 local authorities, along with five national stakeholders participated in qualitative interviews (see Table 1). The majority of local authority representatives were female (67%). The 40–50 age group was most common (47%), as was the Yorkshire and the Humber region (53%). Participants generally worked in Benefits (20%) or Children’s Services (20%), though departments varied. Participant roles varied considerably, though the majority worked in a management role or above (80%). Stages of implementation were categorised as ‘implementing’ (33%) denoting any local authority that was setting up auto-enrolment at the time of the interview, ‘pilot’ (7%) referring to an auto-enrolment pilot processes within a subsection of selected schools, ‘implemented’ (40%) referring to local authorities that had implemented auto-enrolment in the previous 1–2 years and ‘business as usual’ (20%) denoting those that have had auto-enrolment for several years. Table 2 provides further detail by area. Given the low number of national stakeholders who took part in interviews, demographic characteristics have not been shared to protect anonymity.

**Table 1:** Demographic characteristics of interviewed local authority participants. Percentages may not equal 100% due to rounding.

Variable		N = 15	Percentage (%)
***Gender***	Male	5	33
	Female	10	67
***Age***	20–30 years	1	7
	30–40 years	2	13
	40–50 years	7	47
	50–60 years	4	27
	>60 years	1	7
***Location (region)***	North East	1	7
	North West	1	7
	Yorkshire and the Humber	8	53
	London	5	33
***Department***	School Admissions	1	7
	External Consultancy Service	1	7
	Improvement and Change	1	7
	Benefits Department/Services	3	20
	Children’s Services	3	20
	Public Health	2	13
	Finance and Governance	1	7
	Insight, Transformation and Performance	1	7
	Adult Social Care	1	7
	Adults, Health & Wellbeing	1	7
***Occupation***	School Admissions Manager	1	7
	External Consultant	1	7
	Project Manager	2	13
	Benefits Manager	3	20
	Head of School Support	1	7
	Public Health Manager	1	7
	Senior Business Analyst	1	7
	Programme Director	1	7
	Insight and Delivery Lead	1	7
	Project Coordinator	1	7
	Head of Public Health	1	7
	Consultant in Public Health	1	7

**Table 2:** Local authority sample overview.

Local authority identifier	Region	Department	Stage of FSM auto-enrolment**
LA01	Yorkshire and the Humber	School Admissions	Pilot
LA02	London	External Consultancy Service	Implemented
LA03	Yorkshire and the Humber	Improvement and Change	Implemented
LA04*	London	Children and Adults Services	Implemented
LA05	Yorkshire and the Humber	Benefits Department	Implementing
LA06*	South West	Public Health	Implementing
LA07	London	Children’s Services	Implemented
LA08*	East of England	Admissions and School Travel	Exploration
LA09	Yorkshire and the Humber	Adult Social Care	Implemented
LA11	Yorkshire and the Humber	Children’s Services	Business as usual
LA12	Yorkshire and the Humber	Public Health	Implementing
LA13	London	Children’s Services	Implemented
LA14	London	Finance and Governance	Implemented
LA17	London	Insight, Transformation and Performance	Implementing
LA19	North East	Adults, Health & Wellbeing	Implementing
LA20	North West	Public Health	Implementing
LA21	Yorkshire and the Humber	Benefits Department	Business as usual
LA22	Yorkshire and the Humber	Benefits Services	Business as usual

*Denotes local authorities that did not participate in an interview but provided documents for analysis.

**For local authorities that provided documents but did not participate in an interview, auto-enrolment stage was based on the last document provided by the local authority.

Throughout this section ‘participant’ refers to interviewed local authority stakeholders and national stakeholders.

### Documentary analysis

Table 3 provides a breakdown of the types of documents retrieved and analysed. The majority of documents were categorised as general correspondence (35.9%), followed by auto-enrolment updates (24.7%) and workshop notes (13.4%).

**Table 3:** Category breakdown of documents included in the documentary analysis.

Document type	Number analysed (N = 142)
FSM auto-enrolment implementation/data workshop notes *(attended by local authority stakeholders, commissioned consultancy services and members of the FixOurFood team)*	19
Newsletters *(e.g., briefings to parents/residents)*	3
Meeting minutes *(e.g., briefing meetings with the DfE, regional FSM meeting minutes)*	15
General correspondence *(e.g., emails/letters between local authority and government departments)*	51
FSM auto-enrolment updates *(from specific local authorities)*	35
Approvals *(e.g., DPIA approvals from information governance teams)*	6
Other documents/reports *(e.g., MoU, process maps, guides, briefing documents)*	13
***Total***	142

### Thematic framework

Results from interviews and documentary analysis are presented based on framework constructs followed by themes. Seven themes were identified through analysis of the CICI constructs matrix, described within this section: (1) context and setting, (2) interest, exploration and planning, (3) implementation, (4) capacity and resources, (5) support and collaborations, (6) data processes and (7) information governance and legal compliance. Text within square brackets indicate documents from the documentary analysis, which are presented contextually under the appropriate theme.

### Processes, elements and agents required to set-up FSM auto-enrolment

#### Setting: Instability, population and objectives.

All participants reported varying levels of deprivation and poverty within their area. In some, this was relatively new following a period of economic instability. In others, poverty was a longstanding problem which was exacerbated by economic instability, which highlighted the need for local authority intervention. In response, local authorities investigated various opportunities, with FSM auto-enrolment emerging as a potential solution to support residents and increase food security.

*“Poverty is something that’s always been around in this country and absolutely within LA19, but… it’s just been brought into a sharper focus in the last year… it’s been hitting a lot more [people].”* (LA19)

*“With [the] cost of living crisis and people… struggling, there’s a lot of food insecurity. So… it’s about identifying those families who are… [entitled] for free school meals who need that support. And a lot of them do.”* (LA12)

Alongside the need to improve food security, one participant reported an increase in families requiring financial aid and highlighted their role in preventing residents from getting into debt. FSM auto-enrolment was perceived to increase financial security, as families would no longer be responsible for providing meals.

*“So, people that have never… had to think too hard about choices in terms of budgeting, or cutting back on certain things, or going without, are now having to make those choices… with regard to… mitigating the impact and trying to not get into too much debt...”* (LA19)

With circumstances changing due to economic instability, several participants reported an increase in the number of children entitled for FSM, ultimately motivating local authorities to consider auto-enrolment as an intervention.

*“We’ve seen a huge rise in FSM from about 17% in 2017… [to] 28.5% at the moment… That’s a really big rise in a short period of time and a lot of children.”* (LA07)

While economic instability impacted households throughout England, it was participant knowledge of their population that furthered interest in FSM auto-enrolment. Several participants reported higher than average levels of deprivation, unemployment and FSM eligibility. In one area, FSM eligibility was thought to be considerably higher than the national average. In another, a participant reported the highest level of FSM eligibility on record, having observed a marked increase over recent years due to the cost of living crisis.

*“We are a deprived area with a lot of unemployment, one of the most deprived areas in the country.”* (LA05)

*“LA02 has some of the highest… means tested free school meal eligibility in London.”* (LA02)

*“We’re at the highest level of FSM we’ve ever been… without a doubt there is [sic] pressures from cost of living, etc.”* (LA07)

Several participants highlighted issues related to inequalities during interviews, describing the characteristics of pupils registered through FSM auto-enrolment, which were verified through email correspondence. Further exploration of FSM auto-enrolment in other areas revealed additional inequality-related barriers, including cultural and language barriers which affected the current parent/carer application-only registration method. Several participants also reported a diverse range of languages within their area, with a notable percentage of families who had not self-registered as speaking English as an additional language. In the context of FSM registrations through auto-enrolment, several participants highlighted a greater proportion of registrations from families speaking English as an additional language, compared to the proportion in the self-registration group.

*“Out of that group [auto-enrolled pupils], 79% of them came from Black, Asian or multiple ethnicity backgrounds... 50%... didn’t speak English as a first language… 89% [were]... from lone parent families.”* (LA14)

*“67… [pupils that] came through were… unsurprisingly from more disadvantaged cohorts, higher than our general population level. So… there’s clearly something there about culture, language or something we’re not getting.”* (LA07)

*“What really stands out for me is how this… clearly demonstrates that the application-only approach disproportionately disadvantages our Black, Asian or Multi Ethnic population [and] how our ability to use this data can correct this inequity.”* (LA14 [email])

Overall, high rates of deprivation, unemployment and FSM eligibility, in the context of economic instability, emphasised the need for local authorities to support disadvantaged residents.

Driven by the need to improve the financial and food security of residents, participants continued to learn more about FSM auto-enrolment through presentations and FixOurFood webinars. Ultimately, this led local authorities to implement auto-enrolment as an intervention that sat within local programmes that could reduce inequalities on a local level. In some areas, FSM auto-enrolment was aligned within current local authority strategy priority areas.

*“It [FSM auto-enrolment] came to our attention through… FixOurFood, the head of public health was really keen to push this forward under… the [local authority programme].”* (LA03)

Often, the decision to implement FSM auto-enrolment came from senior leaders, who recognised the potential benefits of the programme, and how this could meet the needs of their population.

#### Implementation context: Interest and perceptions.

The majority of participants emphasised the need for continued internal leadership support, as this permitted implementing stakeholders to acquire the necessary resources and support from other teams to take the programme further.

*“Definitely the senior members of the CMT [Corporate Management Team] were crucial to this [FSM auto-enrolment] happening and being successful, definitely, because as soon as we hit a hurdle… they would be... advocating for this to go through.”* (LA03)

The degree to which senior leaders were involved varied, though generally, leadership support was either a facilitator, which allowed implementation to progress efficiently, or was an essential component, without which implementation could not have occurred. Both interviews and documentary analysis highlighted the importance of leadership support.

*“Having a lead member and director of children’s services who is encouraging staff to move forward with auto-enrolment implementation has allowed the process to move forward more quickly.”* (LA07 [workshop notes])

*“Me and [name 1] weren’t of a responsible level that we could make the decisions.”* (LA11)

*“The fact that the process requires many teams and a council to work together, often for the first time, dealing with complex issues, where there is clear governance and strong sponsorship from senior leaders in the council, we have found that any barriers and blockers are able to be resolved.”* (NS01)

Leadership involvement also helped distinguish FSM auto-enrolment as a priority, which helped mitigate team capacity concerns while giving staff the permission to bring teams together and progress implementation.

*“Some of the staff… were more, ‘Oh, this can’t be done. We haven’t got that capacity’.”* (LA03)

*“It’s because I’ve got the seniority in the organisation. When you just say we’re doing something people were just like, ‘Yeah, alright’... Whereas I think in [other local authorities] they had a data team that were a bit more junior and… nobody quite had permission to just say, ‘Yeah, let’s go and do it’.”* (LA14)

Initial perceptions toward FSM auto-enrolment varied. In some areas, there was scepticism as to whether auto-enrolment was necessary, which may have been related to the general risk tolerance of participants or the local authority overall. However, both interviews and analysis of documents revealed that, as participants began to observe the success of the programme (both within their area and others), they felt the fear of risk subside.

“*People talked about those councils that are risk averse, actually, just having access to this information of what we’ve been doing and the success that it’s brought will start to remove some of that fear*.” (LA07 [workshop notes])

*“There was quite a lot of… initial scepticism… It didn’t seem to a lot of departments that it was something that needed to be fixed. And also a lot of schools I think also felt that they had pretty good systems in place… but obviously for a lot of schools it has proven to bring in a lot of funding.”* (LA02)

*“It didn’t feel achievable and then felt very achievable.”* (LA14)

Other local authorities were optimistic from the beginning. Several participants reported changing attitudes throughout implementation, particularly once specific barriers were overcome.

*“At the time members were really keen, my managers were really keen, and it [FSM auto-enrolment] was just seen as a bit of a win-win really.”* (LA22)

### Early stages of implementation

Several participants undertook essential steps early in the process, though the order in which these steps were undertaken differed between areas. These steps included (1) gaining initial approval from senior leaders (often implied if the ask came from a senior manager), (2) identifying those with relevant expertise and setting up a cross council working group, (3) identifying the key responsibilities of those within the working group and finally (4) producing and gaining DPIA and business case approval.

In some areas, additional steps were taken to pilot the data matching process, which allowed local authorities to estimate how many children would be identified and, therefore, the costs and benefits of the process implemented at scale. Piloting also allowed stakeholders to plan for the potential financial pressure caused by delays between identifying pupils and schools receiving funding, which local authorities occasionally had to offset.

*“If we do that kind of trial run with the data and it comes back and says, ‘Oh, yeah, we think there’s 2,000 children here who are… [entitled],’ then we would have to rethink because we couldn’t afford to support and offset that kind of gap that that would leave in the school budgets.”* (LA20)

Irrespective of the sequence in which participants carried out these steps, a common theme throughout was the importance of coordination and cooperation between individuals and teams across the local authority.

Once senior leadership approval was obtained, the next step was to identify the stakeholders responsible for FSM auto-enrolment implementation. As this was a new process in most local authorities, several participants highlighted stakeholder coordination as an important step.

*“We sort of sat down one to one and said, ‘Right, what’s your role? What’s my role? What am I doing? And what are the key things we need to do and who’s going to do what?’”* (LA12)

In one area, this culminated in a task and finish group spanning several departments. In another, project delivery teams remained small, as those within the team had sufficient authority to proceed with implementation with less involvement from other departments. Several participants reported that bringing people together was one of the more time consuming tasks.

“*The planning and the meetings and trying to get stakeholders on board, that was the time-consuming element of it.”* (LA03)

For those responsible for team coordination, the way in which the project was framed was essential to ensure support from all stakeholders (i.e., by discussing the intended benefits of the programme). This was particularly relevant when engaging with information governance colleagues, who were often solely responsible for approving various steps in the process.

Several participants highlighted the importance of consulting information governance colleagues for guidance and to check legal compliance regarding data processing activities. In some areas, this was a relatively straightforward process and gave stakeholders the confidence that what they were doing was legal.

*“You want to have that surety about where you’re standing [legally].”* (LA19)

Conversely, in other areas, information governance considerably slowed implementation, as those implementing the programme had to justify data processing activities and confirm that all alternative options had been exhausted before considering FSM auto-enrolment.

“*Once we’d explained that to information governance… [that] we’d tried this and there really wasn’t another way around it, we just had IG [information governance] on board.”* (LA11)

Participants shared how pushback from information governance colleagues drove them to reconsider the programme and how they could progress, which subsequently relied upon the decisions of a small number of senior leaders.

*“I spent numerous hours with the information governance team and the legal team to try and iron this out... we flagged two major risks… one about sharing data, one about the Education Act… it went to our CMT [Corporate Management Team], and then it went to the chief exec, and they all agreed that… the benefits outweighed the risks and they were happy to take those risks on to do the project.”* (LA03)

#### Full implementation.

**Fig 2:**
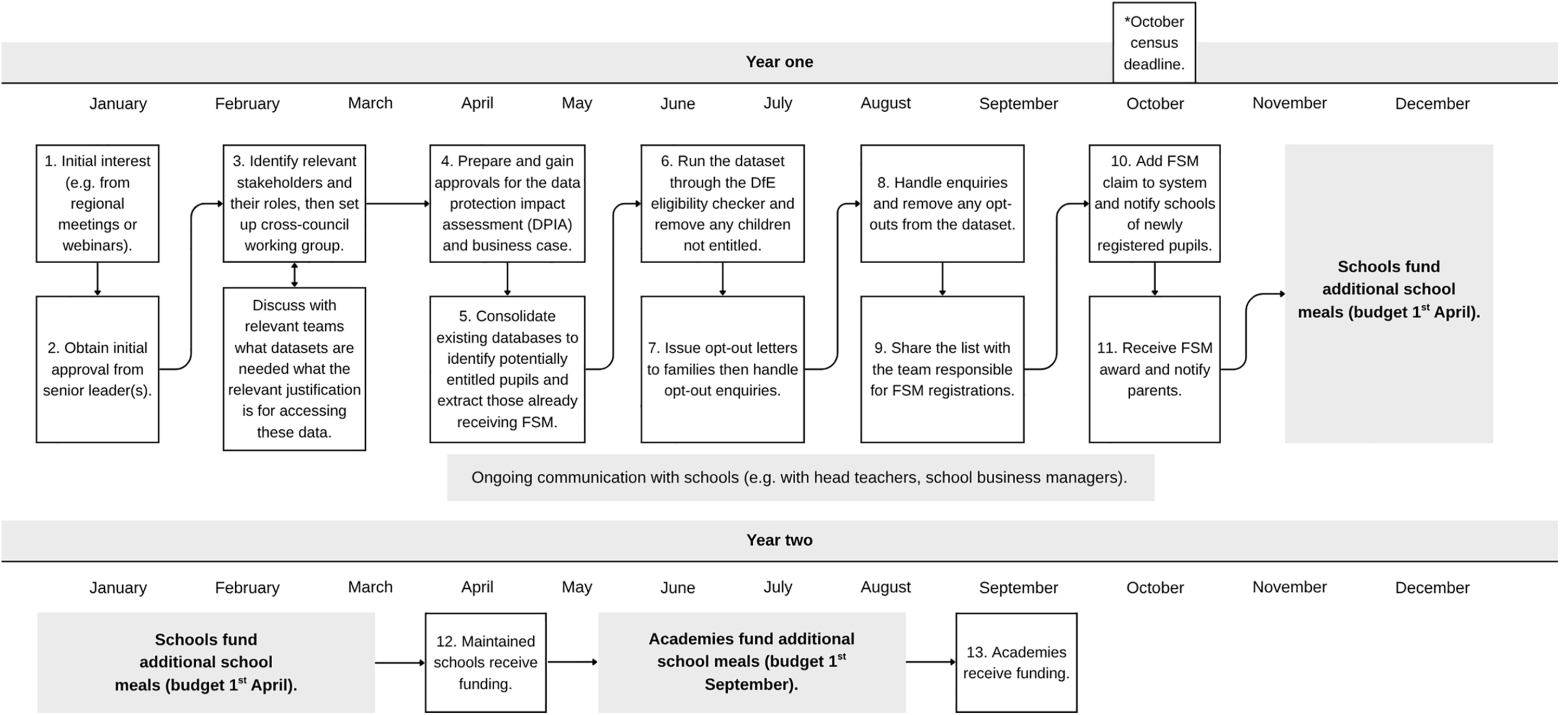
Free school meal auto-enrolment process flow. Implementation timescales are not representative of all local authorities and vary considerably based on barriers and facilitators. *The October census (also referred to as the Autumn census) is a round of statutory data collection from schools that includes data such as free school meal eligibility, absences and special educational needs.

Participants in areas that had implemented FSM auto-enrolment reported that implementation was intensive and contained several stages detailed in the following sections. Fig 2 below provides an overview of the FSM auto-enrolment process.

Depending on the type of data being used, the order in which initial data matching was undertaken varied, relating to existing consent to use different data sources. If participants used council tax or housing benefit data, data matching was used in the beginning to identify and remove those already receiving FSM. This was then followed by writing to parents/carers to give them the opportunity to opt out. In instances where local authorities had deemed consent from parents, participants were able to finalise the list of pupils not receiving FSM more quickly and issue opt-out letters.

“*Because we had deemed consent from parents filling in application forms… we could… extract those people already receiving free school meals [and]… finalise our dataset.”* (LA02)

*“Revenues and Benefits sent the data across… people that were claiming council tax and housing benefit. And we cross-matched that data with the existing free school meals claimants so we could eliminate those… Then we sent out those [opt-out] letters.”* (LA03)

If universal credit data was used (i.e., data identifying families in receipt of means-tested, universal welfare support), several participants reported sending opt out letters in the beginning.

*“The difference in the universal credit data… [is that] we started with the opt out process… because we didn’t have… deemed consent [from parents] to process or to check the eligibility or register the child, so [parents]… needed their… opt out opportunity.”* (LA02).

Irrespective of the order in which datasets were consolidated, all participants offered families an opportunity to opt out. Despite numerous families being offered this opportunity, the numbers opting out were either very low or non-existent.

*“There was one opt-out, they only said they don’t want to be enrolled in free school meals when they replied.”* (LA17)

*“And massive, massive support from parents for this programme, which probably reflects the fact that very few parents have also opted out.”* (NS05)

Of those who opted out, various reasons were given, but mostly related to a lack of understanding of FSM eligibility or the auto-enrolment process. One participant reported four ‘opt outs’ in their area due to home-schooling, and another participant cited a food allergy as the reason one family opted out. One father misunderstood the opt-out letter, not realising that eligibility depends on the parent the child lives with..

*“[The father] thought that the letter sent to him was in error… the parents were divorced and… the father didn’t qualify for benefits, and therefore he thought that his child did not qualify for free school meals.”* (LA13)

Once families that wished to opt out were removed, several participants reported running the remaining potentially entitled families through the national government DfE eligibility checking service [42], before sending the list of entitled pupils to the team within the local authority responsible for FSM registrations.

*“We processed this data through the DfE’s eligibility checker to get the final set [of entitled pupils].”* (LA17)

Alongside identifying additional entitled families, participants reported that the eligibility checking process had the unintended consequence of identifying families that were almost entitled and thus may still require support given the current entitlement threshold. As such, one participant reported using this additional information to develop wider local welfare/support policies.

*“The process in itself is worth going through to find those families who are struggling but not… [entitled] as well, because we will build policies around that.”* (LA07)

In areas with close geographical boundaries, several participants underscored the challenges with identifying pupils who live in one area but go to school in another. In these instances, identifying eligible pupils depended entirely on sharing data across boundaries, which revealed inefficiencies in local implementation.

*“The big limitation is doing it on a local authority by local authority basis, that assumes that children are going to school in the borough in which they live, and therefore you need to start sharing data across borough boundaries.*” (NS05)

“Because in [region] we have a lot of cross-border movement… [and we] only hold the data of children in our schools. We don’t hold data of children who… are out of borough and we have an awful lot of movement.” (LA07)

As FSM auto-enrolment implementation progressed, those leading the implementation were required to communicate with several teams and organisations. Internally, several participants highlighted the need to regularly update stakeholders on progress. Several participants also emphasised the importance of frequent communication with schools, which allowed local authorities to identify pupils that had recently moved into the area.

*“Quite a cross council kind of push. And through that period just going back to brief directors on where we had got to, brief members on where we had got to. And so, yeah, there was a bit of comms involved in just keeping everyone on side.”* (LA07)

*“We can only automate those that we know about. So schools have very much become the partner in this… and we have developed good relationships.”* (LA22)

In addition, the majority of participants shared their knowledge and experiences with other local authorities, occasionally through FixOurFood webinars, which were attended by local authority representatives at various stages of implementation. During these workshops, stakeholders discussed barriers and solutions with their peers and members of the FixOurFood research team. Several participants also emphasised the importance of receiving support from those further along in the process.

*“We’ve found through the FixOurFood meetings that although our neighbouring boroughs are at the start of their process, we’re really happy to work with them, we’d like to form some kind of data sharing cluster with them, which could then be spread across London.”* (LA13)

*“The fact that they [other local authority] are so much further down the line to be perfectly honest, and [name] is so willing to help. The [FixOurFood] toolkit and obviously the co-production and collaboration with other local authorities has definitely helped.”* (LA01)

By maintaining internal and external communication channels, those implementing FSM auto-enrolment could keep internal stakeholders engaged, learn from experienced local authorities and identify additional pupils throughout the year. Similarly, by maintaining relationships with schools, local authority stakeholders could receive feedback specific to the regional context in which local authorities were implementing auto-enrolment. For example, one participant highlighted the need to translate communications to various languages to meet the needs of their population. Another participant reported that schools within their area liaised with parents on their behalf, as schools would likely have greater knowledge of the family situation. This relationship with schools led to a greater number of letters reaching the intended recipient.

“*We were speaking to schools throughout autumn, we got some feedback from them about how to implement, including things around getting letters translated into different languages that are spoken across the borough.”* (LA13)

### Barriers and facilitators of FSM auto-enrolment implementation

#### Barriers – capacity, governance and data processing.

Several participants reported that they were initially concerned they lacked the resources to implement the programme, particularly regarding staff workload, with one participant reporting the impact of recent financial difficulties and how this hindered their ability to bring in additional support to increase capacity.

*“Obviously their [stakeholder] nervousness around capacity and the volumes of the work… ‘It won’t work and we won’t have time, we won’t have the capacity’*.” (LA03)

*“The main issue has been the resourcing of the project… A few years ago, there might have been money to be thrown at it in terms of project teams, etc, we’re not in that place anymore, we’re in a very different financial position.*” (LA01)

Whether staff capacity was a barrier depended on current workload, or if a specific role existed to lead implementation. If a role did not exist, implementation relied on the support of additional teams or external consultancy services, rather than creating a new role solely for auto-enrolment.

*“We are… an additional resource to try these things [additional projects]… that’s the nature of our roles.”* (LA14)

Several participants reported variation in the ability of local authorities to undertake FSM auto-enrolment from a capacity perspective, both now and on a recurring basis as workloads (and thus stakeholder capacity) vary over time.

*“I think some local authorities still don’t necessarily have the capacity for this kind of work, and every local authority is quite different and has different funding and capacity available to them*.” (NS02)

Several participants highlighted that FSM auto-enrolment was, and could be, impacted by the financial context in which the programme was implemented. For instance, one participant expressed concerns around implementing the programme in areas of high deprivation, where they could identify a large number of children. In these areas, schools could face financial difficulties, as the funding for newly identified pupils may not arrive until the following year.

“*The financial strain of paying for those… extra school meals every day between October and April next year wholly sits with the school and their current budget.”* (LA20)

To alleviate the potential financial pressure on schools, this local authority provided interim financial support. This participant also emphasised the importance of using the October census date (See Fig 1) as the deadline to ensure the correct funding allocation and timely receipt of funding, eliminating the need for local authority intervention.

*“The cut-off date was the 5th [October] this year. If we had registered people on the 6th then the school is legally obliged to provide that free school meal, but… funding would not be in the budget until April.”* (LA20)

The financial position of the local authority influenced several aspects of implementation: (a) whether the local authority could support schools as an interim measure, (b) whether the local authority could commission external services to manage the project and (c) whether stakeholders would be willing to undertake new work, in the context of recently increased workloads caused by staff redundancies.

*“Another economic factor at local council level is the ability of the council to be able to provide resource to project manage the opt out auto award process.”* (NS01)

*“The local authority is bankrupt, you know, we’re going through multiple teams across the council, having to go through budget cuts, staff redundancies, all those sort of things which presents additional pressures to the local authority, which directly and indirectly impacts on whether people want to take on new work.*” (LA09)

Although FSM auto-enrolment can increase future funding for schools via pupil premium, one participant highlighted that auto-enrolment does not bring in any income directly to local authorities (where the majority of implementation tasks lie). This view was shared by another participant, who suggested that ‘resourcing costs’ could impact how local authority stakeholders engaged with the process. In one area, as school meal budgets were devolved to schools, schools procured their meals from different sources, and so the cost of the meals varied. Consequently, government funding may not cover the full cost of the meal (currently £2.61 for most of England), as one participant suggested, and schools would be required to make up the difference, which could be particularly problematic in heavily deprived areas where many pupils could be identified. Further, delayed payment could impact some schools more than others.

*“This process [FSM auto-enrolment] does not drive income for the council at all. It may drive income for schools through increased free school meal registration and… [pupil] premium funding down the line, but the expenditure of the process is not aligned to the income in an immediate cost benefit.*” (NS01)

*“Resourcing costs… whether… there’s been an investment of budget in managing that programme, how many staff hours it might take… I think there’s been some reticence to engage with it [FSM auto-enrolment].”* (NS05)

*“LA20… devolved school meal budgets to the schools themselves… So, every individual school… they commission and procedure their own school food… [and] school meals range from £2.30… to £3.25 per meal… So schools were still subsidising all the free school meals for the children who were already registered.”* (LA20)

Several participants found sourcing welfare data to be challenging, and experienced that data controllers were wary of sharing data because of confusion relating to the legalities of data use.

*“There were some sort of internal barriers around hesitancy in terms of how we use up people’s data. Is it a change of use? Is it, are we complying with GDPR [General Data Protection Regulation]?”* (LA13)

One participant revealed considerable difficulties engaging the welfare stakeholders involved in data matching, due to the perception that all entitled children were already registered. This participant expressed frustration that this assumption persisted despite data to the contrary, which led to substantial delays.

*“The main barrier we had was literally engaging with our revenue and benefits team, and that was predominantly driven by, like I said, there was a bit of arrogance around thinking that there [wasn’t] a problem…”* (LA09)

Several participants also experienced barriers when accessing universal credit data from the national government Department for Work and Pensions (DWP). However, in one instance, the local authority had an information sharing agreement with the data controller (DWP), which gave stakeholders the confidence to use the data without seeking explicit permission.

*“I know that some local authorities have raised a bit of an issue about using data that’s come direct from DWP… because we haven’t directly collected it… [but the information sharing agreement] basically says that we will use this information for welfare purposes, and as far as we’re concerned at LA11, free school meals is a welfare purpose.”* (LA11)

On several occasions, database access depended on the type of local authority, which, for those undergoing a restructuring process, changed the permissions for data access. For other, non-unitary councils, this meant obtaining the information from the data controller (the DWP or other councils).

*“[The] main barrier so far is being a non-unitary Council so we don’t hold the datasets required and would need to gain the information from DWP or local borough and district councils.”* (LA08)

“*Formally we were two tier and so… [we need to be] quite sure of the potential consequence of DWP removing the council’s licence to access Searchlight to administer our localised benefits system.”* (LA20)

Once stakeholders had sourced the data, there were further barriers relating to data consolidation and processing, which subsequently caused delays and increased costs, as stakeholders commissioned additional support to extract the data from different sources.

*“We don’t even have a database which would connect some of these key data sources together… there is always waiting time and there is a charge associated with it for developer time to write the script that extracts the data.”* (LA17)

*“The requirement to match together different types of databases is flawed because there will always be an element of fuzzy matching or inaccurate matching of data.”* (NS01)

As participants faced various data-related barriers, this led some to consider whether they could re-use data for the purpose of FSM auto-enrolment. However, stakeholders felt that they had received inconsistent responses from the national government regarding permissions to use specific datasets. Subsequently, these inconsistent responses caused further stakeholder apprehension and delays. In one instance, a participant contacted their local Members of Parliament for clarification, but did not receive a clear response.

*“I know… [name] wrote to the DWP and applied for a reuse of data and then they were rejected…”* (LA19)

“*There is no consistent application of legal basis… and that has caused significant variation in a council’s accessing the benefits data source in order to be able to identify entitled families.*” (NS01)

*“I drafted those written questions recently and suggested them to those MPs [members of parliament] and naturally was eagerly awaiting the answers in the hope that we would see in very clear, stark, explicit language that local authorities were free to use data… but there was still that caveat around… data being used within the boundaries of existing legislation.”* (NS03)

In some instances, stakeholders would not begin exploring FSM auto-enrolment, due to the fear of pushback from the national government, as highlighted by one participant: “[Local authorities] *are not even prepared to begin looking because of fears of reprisal from the national body*” (NS01). Of all the barriers to implementation, this participant suggested that data concerns were the barrier that stopped intent, and thus could not be overcome by sufficient leadership support. Another participant had a similar view, suggesting that implementation ultimately relies on access to the data.

At the time of this research, national government has provided clarity regarding the legality of FSM auto-enrolment; however, during the early stages of implementation, external organisations began to raise legal concerns in some areas, including the lawfulness of FSM auto-enrolment implementation. This led to local authority stakeholder apprehension, as many were implementing the programme for the first time and therefore did not fully understand the legal implications.

*“I think information governance, it’s a scary thing for people and I think it is always a barrier because we are all rightly very worried about doing the wrong thing.”* (LA14)

*“We’d started hearing from [name] in [organisation] that the DfE had asked them to stop publicising it [FSM auto-enrolment].”* (LA11)

Interviews and documentary analysis showed that stakeholders supported one another throughout implementation. Those that were contacted by government organisations shared their concerns with others, which increased apprehension in other areas that had not been contacted by government organisations.

*“We’ve already had issues with the IG team and the legal team anyway and they were nervous about this project going forward, getting this [feedback from other local authorities] has just added to that nervousness, so I’m unsure [whether this will impact future implementation].”* (LA03)

*“The push back and inconsistency at the national level makes us feel like we are doing something wrong or illegal when in fact all we are trying to do is support children to receive a fundamental necessity of food to which they are perfectly entitled to.”* (LA20 [email])

One participant suggested that legal confusion may have arisen as DWP guidance could be open to interpretation. Proceeding with implementation then, could be influenced by risk tolerance, where some would seek clarification from the DfE and DWP, and others would be more confident in their legal position.

“*The difficulty is that DWP are not precise enough in their wording… [it] is not specific enough in the legislation or the guidance that we are issued with as a local authority*.” (LA20)

Several participants reported that adversity to risk generally stemmed from information governance teams and related to the opt out and eligibility checking process. Additionally, information governance stakeholders raised concerns relating to the legal basis for data processing and General Data Protection Regulation [GDPR].

*“Obtaining details of benefits a family receives without their knowledge (because they haven’t made a request) may breach other rights… [and] having an opt out option does not sufficiently ensure that individuals are given the opportunity to withhold consent to such information about them being shared.”* (LA06 [email]).

Several participants highlighted that the decision to proceed with implementation, despite resistance from information governance stakeholders, often relied on senior leadership support.

*“We had a lot of sway from above… and I think if we hadn’t have had that support… when we did hit the barriers of the information governance and the legal information, I think that we probably would have faltered at that point.”* (LA03)

*“Sometimes you feel like there’s a bit of reluctance… the legal stuff and our data governance team, there’s been a few concerns coming from them as well… My manager [name]... thinks it’s a great idea [FSM auto-enrolment]... So, it’s good to have that support.*” (LA12)

As information governance was a considerable barrier to implementation, one stakeholder shared this with their senior leadership, who consequently initiated discussions with national government representatives, aiming to clarify the government’s legal position.

*“I have taken this up with our Director of Transformation who is trying to unblock this nationally. National government are looking at areas where national processes are creating barriers to local government work and he is keen to get this resolved.”* (LA12)

#### Facilitators – internal and external support and collaborations.

Participants recounted three overarching facilitators that helped them overcome barriers: implementation support, external collaborations and document sharing.

Several participants emphasised the role of bringing relevant stakeholders together early in the process, which was often achieved by gaining support from senior leaders with the authority to mobilise teams. Guidance on which people needed to be involved came from other local authorities.

*“Make sure that you get a really good coverage of people at the right level from the right services… [somebody from] a legal perspective… information governance… And then clearly you need… teams that are actually doing the job, so your revenues and benefits team, your customer services team… “* (LA19)

*“In terms of the whole approach, it was very good that we had support from the top... I think that helped a lot in terms of mobilising people and steering this from the very beginning.”* (LA17)

While senior leaders were involved in bringing teams together, others were critical in overcoming barriers, with one participant highlighting the importance of councillor engagement to influence teams.

*“This particular elected member, as well as working with us, is also highly respected as an elected member within the rest of the council and across all other elected members, so that also helps to have a person that is influential politically.”* (LA09)

Two participants found that having a small project team helped, as this meant that they did not have to rely on other people/teams.

*“We were able to get things done quite quickly. There [weren’t] too many people that we were relying on.”* (LA13)

*“We’re a small team. So we can kind of have those [FSM auto-enrolment-related] discussions quite quickly.”* (LA01)

Communication and knowledge sharing with other experienced local authorities also facilitated implementation, particularly when there were legal challenges, as this provided initial guidance followed by reassurance that stakeholders were implementing the programme correctly.

While support from internal colleagues facilitated implementation, local authorities were limited by their FSM auto-enrolment knowledge. As such, the majority of participants sought process guidance from experienced local authorities, many of which led to the development of new external collaborations and sharing of relevant documents.

“*We did have a conversation with [local authority] and we had a conversation with other people about the processes they went through and the challenges that exist.*” (LA09)

*“Collaboration with other local authorities has definitely helped… we are kind of all learning from each other and more than happy to share best practice or… lessons learned… that has been key to it.”* (LA01)

Often, implementation support came from FixOurFood webinars, which gave those implementing auto-enrolment the opportunity to receive tailored feedback from peers at a similar stage of implementation.

*“Through early last year it was about… attending webinars, getting that information, trying to have a better understanding of what the issues are, what we might need to think about... tapping into the regional support that’s there.”* (LA12)

As local authorities connected through FixOurFood webinars, some learned that neighbouring authorities were implementing FSM auto-enrolment, and used the opportunity to establish relationships with those who understand local challenges. For example, local authorities in one region used this as an opportunity to share information to better address local challenges, which ultimately led one local authority to formalising this arrangement through a data sharing agreement. By sharing data, this allowed local authorities to identify and check eligibility for children whose address had changed and check whether they were entitled for FSM.

*“The nature of London is that boroughs are quite small, people don’t live within their borough boundaries, people cross over and some don’t actually realise that they’re doing it, and so being able to share information across two boroughs was helpful.”* (LA13)

In situations where local authorities needed guidance, they made use of an available FixOurFood FSM auto-enrolment toolkit, which included templates and example documentation from experienced local authorities.

*“Our process was very much influenced by the toolkit that’s in place as well as the [local authority] and [local authority] process.”* (LA02)

Further, if and how stakeholders would use the FixOurFood toolkit depended on their stage of implementation, with some having implemented FSM auto-enrolment before the toolkit was developed. In these situations, stakeholders would use the toolkit retrospectively to check that they had included all the essential steps. Similarly, those earlier in the process found that the toolkit provided an overarching view and critical steps to implementation and in some instances, answers to specific questions.

*“The toolkit does reflect how we’ve done it, and certainly what our intended path is going forward, it lines up very well with the toolkit.”* (LA20)

*“I think what the toolkit does is it shows you no matter where you are in the council… there are some parts that you can’t do it without.”* (LA17)

Several participants found that example documents provided by experienced local authorities within the toolkit helped facilitate implementation, as this gave local authorities a starting document which they could adapt to their local context. The documents provided were considered essential to implementation, and included example legal documents, DPIAs and privacy notices.

*“We sort of followed [local authority’s] process. We got the information from them. We got the letters from them. We tweaked the letters to suit us.”* (LA03)

## Discussion

This study explored local authority implementation of FSM auto-enrolment processes, considering implementation strategies, barriers and facilitators. Our results reveal considerable variation in implementation processes and barriers faced by local authority, particularly concerning information governance and data processing. Despite these barriers, there was a consensus that support and collaborations could overcome obstacles, depending on the support available. Though key themes emerged across local authorities, many of the points of discussion were unique to individual authorities and may be attributed to government decentralisation, whereby the national government delegates power and resources to local authorities [43,44].

Our results revealed multiple points at which stakeholder prioritisation could influence implementation. One key point was support from internal senior leaders (e.g., director of public health, assistant director of education), which participants considered crucial to successful implementation. This finding is comparable to previous research identifying leadership as a key influence on implementation [45–47] and raises the question of whether a lottery exists, whereby leadership interest, or lack thereof, could slow or prevent implementation. This potential variation could be based on: (a) leadership perception of whether residents require support, (b) whether FSM auto-enrolment is considered a priority and (c) whether leaders would persist despite barriers. Alongside leadership perspectives, our results demonstrate how the perspectives of teams and individuals could influence the success of implementation. For example, one local authority explained how they struggled to implement FSM auto-enrolment for almost two years, as essential stakeholders felt it was not necessary. However, once permissions were granted, this local authority shared with us that they identified over 1000 children who were entitled but not registered for FSM. This finding was not exclusive to one local authority and may indicate a wider problem whereby FSM auto-enrolment processes are not initiated based on the mistaken perception that current processes are able to register all entitled pupils.

Our findings also highlighted organisation level influences, such as risk tolerance and openness toward new interventions. Further, as FSM auto-enrolment processes often involved several teams within each local authority, this increased the likelihood of implementation being subject to individual personal/professional interest, risk tolerance and perceived workload. Consequently, broader stakeholder involvement may lead to challenges, such as a lack of interest, risk aversion or perceived high workloads – that may slow or stop implementation. These findings are similar to a recent report published by the Education Policy Institute, that highlighted auto-enrolment as a ‘resource-intensive’ process that requires adequate staff capacity to implement [25]. In addition, previous research [19] exploring implementation of a 4-week lunch menu in Swedish primary schools, highlighted limited capacity as a barrier to implementation [19] Further, research which [48] investigated local authority implementation support provided to local restaurants to implement a ‘Healthier Catering Commitment’, observed similar delivery-related barriers, including difficulties with the additional workload, funding and resource and time intensity [48]. Given that capacity and stakeholder perceptions can impact implementation, this further emphasises the crucial role of senior leaders to agree to project resourcing and unite departments to facilitate implementation.

The financial situation of local authorities also influenced implementation in two ways: (a) the inability to increase capacity and (b) the ability to use available resources to offset gaps between registration of FSM and receipt of funding (often borne through school budgets). Comparably, inadequate resources have been cited as a barrier in previous research exploring local authority process implementation in food-related disciplines [47–49]. Although local authority finances were not explicitly addressed in all interviews, participants highlighted challenges that could be mitigated with sufficient funding, such as limited team resources and a lack of legal expertise. Consequently, financial difficulties could disincentivise implementation at any stage. In particular, in areas with many schools whose budgets are managed by the local authority, and where local authorities expect to identify a large number of pupils. Notably, financial difficulties are not experienced equally, leading to variation in FSM registration and receipt, depending on the financial stability of the local authority. In regions such as London, financial aid was provided by the Greater London Authority to support local authorities through this process [25,50]. However, there are no similar support mechanisms outside of London. Thus, the potential financial burden of identifying many pupils (for example in heavily deprived areas), may not be feasible for some. This situation is exacerbated by the context in which FSM auto-enrolment is implemented, with many local authorities now facing financial difficulties [51], and up to 43% of local authorities being at risk over the next several years, according to the National Audit Office [52].

Alongside stakeholder perceptions and local authority finances, our results indicate challenges with data accessibility, matching and information governance, which also varied widely based on local authority type, risk tolerance and inconsistent feedback from government organisations. These findings further underline the barriers to local implementation. In terms of local authority classification, these findings are likely to be amplified in two-tier authorities (i.e., local governments with two distinct councils with separate responsibilities), where complexities over data ownership are likely to act as a significant barrier to FSM auto-enrolment.

Despite several barriers to local implementation, our results reveal some facilitators, including stakeholder knowledge, collaboration and document sharing, alongside the essential support of senior leaders. In some areas, implementation was facilitated by specific teams which the local authority employed. Unexpectedly, our findings revealed additional benefits to implementation alongside additional FSM registrations and pupil premium funding. Specifically, the eligibility checking process was useful in and of itself, as it allowed local authorities to identify children whose families were marginally outside the FSM eligibility threshold, but who would still require support. This meant that local authorities could create new policies to provide additional support. In the broader context of FSM entitlement, being able to identify struggling families through eligibility checking could be particularly useful given the recent increase in FSM eligibility to include children of families receiving universal credit [7], and the ongoing debate surrounding the FSM eligibility threshold [53]. Similarly, our findings reveal the potential of the FSM auto-enrolment process to access data to reduce inequalities, through the identification of the characteristics of families who have not self-applied for FSM for their children (for example, with higher proportions of families with English as their second language not registered). However, further evidence is required to reverify this finding, as this was not universally discussed.

### Strengths and limitations of the study

This study adds evidence to an under-researched area and explores an alternative FSM registration system in England that is both socioeconomically and politically relevant. As the number of families that require governmental support continues to rise, there is a crucial need to ensure that interventions are underpinned by robust evidence. This study aimed to provide such evidence, and the grounding for future investigation into FSM auto-enrolment as a potential intervention to support disadvantaged families. Secondly, this is an area that is highly policy relevant, recently being addressed by the Education Committee [54] and the House of Lords Food, Diet and Obesity Committee [15]. Alongside socio-economic and political relevance, the findings of this study may extend to international organisations facing challenges with registering pupils or implementing similar programmes to support residents.

On the other hand, the study did not boast a representative sample in terms of geography or local authority structure (e.g., two-tier authorities), and thus findings may not be directly applicable to all areas. Primarily this was caused by the limited number of local authorities undertaking FSM auto-enrolment in underrepresented regions during the recruitment stage, which, to our knowledge, have increased considerably over recent months. Given the relatively smaller number of local authorities implementing auto-enrolment during the recruitment stage of this study, we did not specifically aim to recruit local authorities that had tried and failed at setting up auto-enrolment within their area. However, our sample did provide a detailed account of the varying experiences of local authorities, and included those that continue to experience barriers several years later. Thus, our sample may reveal some of the barriers and facilitators that other local authorities could experience, regardless of geography or local authority structure. Lastly, the recent increase in FSM entitlement highlights a further limitation, as this paper did not explore the impact that an increase in entitlement could have on local authorities implementing FSM auto-enrolment. As such, further research is needed to understand this area.

### Policy implications and future research

Given the substantial blockers to local implementation and the resulting FSM registration lottery, consideration of a centralised approach may be warranted. If the responsibility for identifying pupils was changed to central government, this would overcome many of the persistent data-sharing barriers between local areas. Advocacy in this area has begun, with FSM auto-enrolment being discussed in the Children’s Wellbeing and Schools Bill [55], though it is unclear at this stage whether auto-enrolment of FSM will be included in the UK Food Strategy currently being developed [56]. Such a programme would complement the recently announced increase in FSM entitlement [7]; however, newly announced policies for increasing the eligibility threshold to all children whose family qualifies for universal credit do not include a change in the way that school funding (pupil premium) is provided, which will still only apply to those with an annual household income of less than £7,400. This is likely to create further confusion within local authorities, who will have to identify both those children who meet the new and existing (more restrictive) criteria and only link school funding to the latter. Therefore, while national implementation remains the most efficient solution, in the absence of this, it is essential that local authorities receive support to minimise implementation-related confusion. This may be achieved through explicit data-sharing agreements, to reduce the risk of entitled children being missed by the system. This paper provides first-hand experience of the challenges faced by local authorities in local level implementation of auto-enrolment of FSM. Further research is needed to explore the transferability of auto-enrolment to other contexts, acceptability (to schools and parents) and the quantitative impact compared to the current self-registration system.

## Conclusion

Overall, interviews with local authority stakeholders at various stages of FSM auto-enrolment implementation and national stakeholders highlighted the benefits of the programme, not only to increase pupil registrations and additional funding, but to identify and address inequities experienced by the most disadvantaged families. While local implementation is achievable and may have some advantages (i.e., greater population knowledge), our results suggest that local implementation is reliant on specific conditions to succeed. As conditions such as leadership support, capacity, adequate funding and data access differ by local authority, this raises the matter of a postcode lottery, whereby entitled children are only registered under specific conditions. One solution to the potential postcode lottery could be national government implementation, which would not only make many local barriers redundant, but do so in a way that complements current policies to reduce inequalities in children in England.

### Glossary

**Table:** 

Term	Definition
Combined authority	A type of regional authority that combines responsibilities of smaller authorities to carry out new functions across the region.
County council	A type of two-tier council that is responsible for strategic services such as public health.
Department for Education (DfE)	The UK Government department responsible for services such as education, early years and apprenticeships. In the context of free school meal auto-enrolment, the DfE provides guidance for local authorities and the eligibility checking service.
Department for Work and Pensions (DWP)	The UK Government department responsible for services such as welfare, pensions and child maintenance. In the context of free school meal auto-enrolment, the DWP controls access to universal credit data.
District council	A smaller two-tier type of council, within a county council. Mostly responsible for place-related services such as housing and licensing.
Eligible	Denotes children currently receiving free school meals (the same as ‘registered’ for the purpose of this research).
Entitled	Denotes children who reach the threshold to receive free schools meals but may or may not be registered.
Greater London Authority (GLA)	A type of strategic authority in London responsible for services such as transport, policing and housing.
Registered	Denotes children currently receiving free school meals (the same as ‘eligible’ for the purpose of this research).
Two-tier authority	A type of local government where government functions are split between two types of council: county council and district council.
Unitary authority	A type of local government where one local authority carries out all of the functions of a county and district council.

## Supporting information

S1 Appendix Local authority interview topic guide. Topics in green were used exclusively in interviews with local authorities that were implementing FSM auto-enrolment at the time of the interview.
(DOCX)

S2 Appendix National stakeholder interview topic guide. (DOCX)
