# Supplementary material for: Improving access to free school meals: Evaluating the implementation of free school meal auto-enrolment processes
Source: PLoS One. 2026 Feb 17;21(2):e0339477. doi: 10.1371/journal.pone.0339477 (PMC12912541; doi:10.1371/journal.pone.0339477)
Supplement: S1 Appendix — Topics in green were used exclusively in interviews with local authorities that were implementing FSM auto-enrolment at the time of the interview. (DOCX) [file pone.0339477.s001.docx]

# Local authority Interview topic guide FixOurFood: Free School Meal Auto-Enrolment project

*Thank you for attending today to take part in the interview. I am a researcher at the University of York who is involved in evaluating the implementation of the Free School Meal Auto-Enrolment project. You have been invited to take part in this interview as I would like to hear about your experience of the setting up and rolling out the auto-enrolment and explore your views on what could influence the successful roll out of the project. The interview should last between 30 and 45 minutes.*

*Could you please confirm if you have had a chance to read the participant information sheet?*

*Do you have any questions?*

*I would like to make an audio recording of our discussions as this will help me with the analysis of the data and ensure that we have a full account of everything that is said. After the interview, the interview will be transcribed and any information that could identify you will be removed. I will record what you say but not who says it. Similarly, names of any other people that you mention will also be blanked out or changed so that both you and they can remain anonymous. All data will be stored securely at the University of York. Before we start, can I just confirm that you are happy with that?*

[Begin recording]

*I will now need to take your consent for taking part in the study. This will involve me reading a statement aloud and you will need to verbally state whether you are happy with the statement. This will be recorded and stored as a record that you understand what is involved and are happy to take part.*

[Conduct verbal consent process]

[Stop recording]

*Thank you, I will now begin with the interview questions.*

[Begin **new** recording]

*Firstly, I would like to collect some background information about yourself.*

**Could you please tell me which of the following age ranges you are in?**

**Less than 20 years
20-30 years
30-40 years
40-50 years
50-60 years
Over 60 years**

**What is your job title?**

**How long have you had this job role?**

**How would you describe your gender?**

**How would you describe your ethnicity?**

*Thank you, I would now like to hear about your views of Free School meals and the auto-enrolment process.*

**1. Can you explain your involvement in the roll out of the Free School Meal Auto-enrolment programme?**

- Prompt: Please tell me about your role at [insert local authority name].
- Prompt: What does your role entail in relation to the auto-enrolment programme?
- Prompt: What stage is your local authority at with regards to the roll out of the programme?

**2. If you know of this information, could you please describe your local population and how you feel that your population has impacted on the set up and roll out of the programme? For example,** d**o you think you have a high population who would be eligible for free school meals?**

- Prompt: What do you think is the importance of the programme to your particular population?

**3. How and why did the local authority first become interested in auto-enrolment?**

- Prompt: How did you find about the programme?
- Prompt: What was the initial perception of the programme within your local authority and what do you think influenced this perception?
- Prompt: What influenced your interest in the auto-enrolment programme? (i.e., perceived need, perceived ease of implementing the programme?)

**4a. Could you please describe the process that was followed during the setting up of the auto-enrolment within your local authority?**

- Could you tell me about the workforce required to support the set-up and roll out within your local authority?
- Could you tell me your experience of the governance and data protection processes?
- Could you tell me your experience of working with schools?

**4b. Which processes are you working towards (or do you think you would follow) to set up the auto-enrolment programme within your local authority?**

- Prompt about potential considerations at each stage (Governance and data protection, consolidating data, roll out and engaging with families, registration and notification to schools).
- Prompt: Which teams and stakeholders do you think are (or would be) important to involve in the setting up of the auto-enrolment programme?

**5a. Could you please tell me if you followed the process outlined in the Auto-enrolment toolkit that was based on the process Sheffield had followed?**

- Prompt: Are you aware if your local authority followed a specific approach?
- Prompt: How closely do you feel the process followed the toolkit/ the Sheffield process?

**5b [If Sheffield approach was taken] Could you please describe anything you needed to adapt within the auto-enrolment process based on the needs of your local authority?**

- Prompt: Why were these adaptations needed?
- Prompt about each stage (Governance and data protection, consolidating data, roll out and engaging with families, partnership working with schools).
- How did these adaptations help you with the roll out of auto-enrolment?

**6a. Could you please describe what you think supported you in successfully setting up and implementing auto-enrolment?**

- Prompt about each stage (Governance, consolidating data, roll out and engaging with families, registration and notification to schools).
- Prompt: What support did you receive and how did you find the support?
- Prompt: What was essential to the successful set up and implementation of the programme? (e.g. partnerships, key processes)

**6b. Could you please describe what you think could make it more likely that the set up and implementation of the auto-enrolment will be successful?**

- Prompt about each stage (Governance, consolidating data, roll out and engaging with families, registration and notification to schools).
- What support would you need and from who?

**7a. Could you please describe any barriers or challenges you faced when setting up and implementing auto-enrolment and how you overcame them?**

- Prompt about each stage (Governance and data protection, consolidating data, roll out and engaging with families, registration and notification to schools).
- Prompt: What do you think caused these challenges? (e.g. population characteristics or contextual factors such as policy, legal, implementation strategies)
- Prompt: Would you do anything differently or recommend ways for other local authorities to avoid these challenges?

**7b.** **Could you please describe any barriers you are currently facing or you envisage through setting up and implementing auto-enrolment and how would you overcome them?**

- Prompt about each stage (Governance and data protection, consolidating data, roll out and engaging with families, registration and notification to schools).

**8. [If not addressed in Q6 or Q7] What do you think could impact the year-on-year roll out of auto-enrolment?**

- Prompt: What resources would be needed (i.e., staff capacity, funding)
- Prompt: Which stakeholders or key players would need to be involved and on board?

**9. How could central (national) government support the set up and roll out of auto-enrolment?**

- Is there anything the government is doing or could do to make the roll out of auto-enrolment in your local area more successful? (i.e., national advocacy)

**10. With the knowledge and experience that you have now with setting up auto-enrolment, what advice would you give to someone starting out?**

- Prompt: What should local authorities consider before setting up auto-enrolment? (e.g. who are the key players needed, which processes are essential)
- Prompt: What support should they access?
- Prompt: What are the key barriers to overcome?

***Thank you for answering those questions, that is the end of the questions I would like to ask but before the interview ends is there anything you would like to add?***

*[stop recording]*
