# Supplementary material for: Improving access to free school meals: Evaluating the implementation of free school meal auto-enrolment processes
Source: PLoS One. 2026 Feb 17;21(2):e0339477. doi: 10.1371/journal.pone.0339477 (PMC12912541; doi:10.1371/journal.pone.0339477)
Supplement: S2 Appendix — (DOCX) [file pone.0339477.s002.docx]

# National stakeholder Interview topic guide FixOurFood: Free School Meal Auto-Enrolment project

*Thank you for attending today to take part in the interview. I am a researcher at the University of York who is involved in evaluating the implementation of the Free School Meal Auto-Enrolment project. You have been invited to take part in this interview as I would like to hear about your experience of being involved in the auto-enrolment programme and explore your views on what could influence the successful roll out of the project. The interview should last between 30 and 45 minutes.*

*Could you please confirm if you have had a chance to read the participant information sheet?*

*Do you have any questions?*

*I would like to make an audio recording of our discussions as this will help me with the analysis of the data and ensure that we have a full account of everything that is said. After the interview, the interview will be transcribed and any information that could identify you will be removed. I will record what you say but not who says it. Similarly, names of any other people that you mention will also be blanked out or changed so that both you and they can remain anonymous. All data will be stored securely at the University of York. Before we start, can I just confirm that you are happy with that?*

[Begin recording]

*I will now need to take your consent for taking part in the study. This will involve me reading a statement aloud and you will need to verbally state whether you are happy with the statement. This will be recorded and stored as a record that you understand what is involved and are happy to take part.*

[Conduct verbal consent process]

[Stop recording]

*Thank you, I will now begin with the interview questions.*

[Begin **new** recording]

*Firstly, I would like to collect some background information about yourself.*

**Could you please tell me which of the following age ranges you are in?**

**Less than 20 years
20-30 years
30-40 years
40-50 years
50-60 years
Over 60 years**

**How would you describe your gender?**

**How would you describe your ethnicity?**

**What is your job title?**

**How long have you had this job role?**

*Thank you, I would now like to hear about your views of Free School meals and the auto-enrolment process.*

**1. Can you tell me about your involvement in the auto-enrolment programme?**

- Prompt: What influenced your and your organisation’s interest in the auto-enrolment programme? (i.e., perceived need)
- Prompt: What have your organisation’s activities involved in relation to the auto-enrolment programme? (i.e., advocacy, implementation support)
- Prompt: What have your individual activities involved in relation to the auto-enrolment programme? (e.g. outputs, meetings etc.)

**2. Which organisations have you worked with in relation to auto-enrolment and what have your activities involved? (e.g. local authorities, the Food Foundation)?**

- Prompt: Which collaborations have been important and why?

**3. Could you tell me your views on the auto-enrolment programme?**

- Prompt: Could you explain if you think an auto-enrolment programme is needed and why?
- Prompt: Could you describe what you think are the key potential benefits of the programme? (to families, schools, society)
- Prompt: Could you describe what you think are the key potential limitations of the programme? (to families, schools, society)

**4. From your experience, could you describe what you think could influence the successful roll out of the auto-enrolment programme in other local authority areas?**

- Prompt: What do you think could facilitate the roll out of the programme and why?
- Prompt: What do you think are the barriers to the roll out of the programme and why?

**5. What do you think could impact the year-on-year roll out of auto-enrolment across local authorities?**

- Prompt: What resources would be needed (i.e., staff capacity, funding)
- Prompt: Which stakeholders or key players would need to be involved and on board?
- Prompt: What are the potential barriers and facilitators to the year on year roll out? (i.e., fundings, legal challenges)

**6. What is your opinion of national vs local delivery and what do you think could influence the auto-enrolment programme being delivered centrally at a national level (in other words - not by local authorities)?**

- Prompt: What do you think are the barriers to the programme being rolled out at a national level?
- Prompt: What do you think could facilitate the programme being rolled at a national level?
- Prompt: Who are the key players who would need to be involved in support roll out at a national level?

**7. How do you think auto-enrolment fits with the wider school food system and food programmes?**

- Prompt: Could you explain how the different school food programmes could impact on the roll out of the auto-enrolment programme? (e.g. Universal free school meals)
- Prompt: Could you describe if and how any other school food initiatives could complement or compete with the auto-enrolment programme?

**8. Could you describe what you think the policy recommendations should be for the auto-enrolment programme?**

- From your experience, what recommendations are needed to support the roll out of the auto-enrolment programme both by local authorities and centrally?
- How do you think these recommendations would fit (or compete) with other school food policy recommendations? (Universal free school meals, adapting FSM eligibility criteria, whole school approach to food monitoring, School Food Standards monitoring etc?)

**9. Do you have any other comments about the auto-enrolment programme that we have not yet discussed that you’d like to share?**

***Thank you for answering those questions, that is the end of the questions I would like to ask but before the interview ends is there anything you would like to add?***

[stop recording]
